# Supplementary material for: Palbociclib Induces the Apoptosis of Lung Squamous Cell Carcinoma Cells via RB-Independent STAT3 Phosphorylation
Source: Curr Oncol. 2022 Aug 18;29(8):5855–68. doi: 10.3390/curroncol29080462 (PMC9406926; doi:10.3390/curroncol29080462)
Supplement: Supplementary file 1 [file curroncol-29-00462-s001.zip › curroncol-1827336-supplementary.pdf]

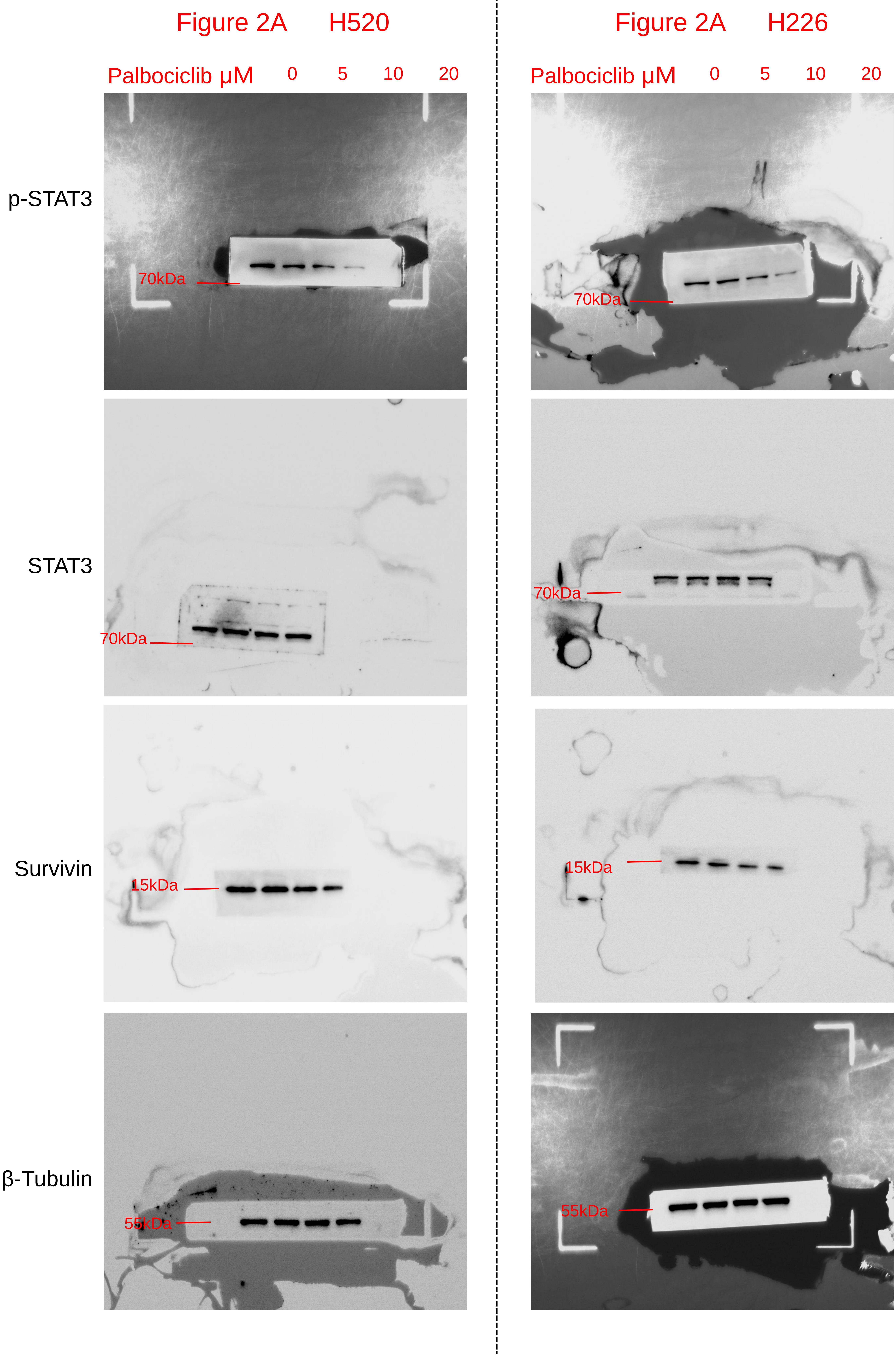

Figure S1. The original image Western blotting of Figure 2A.

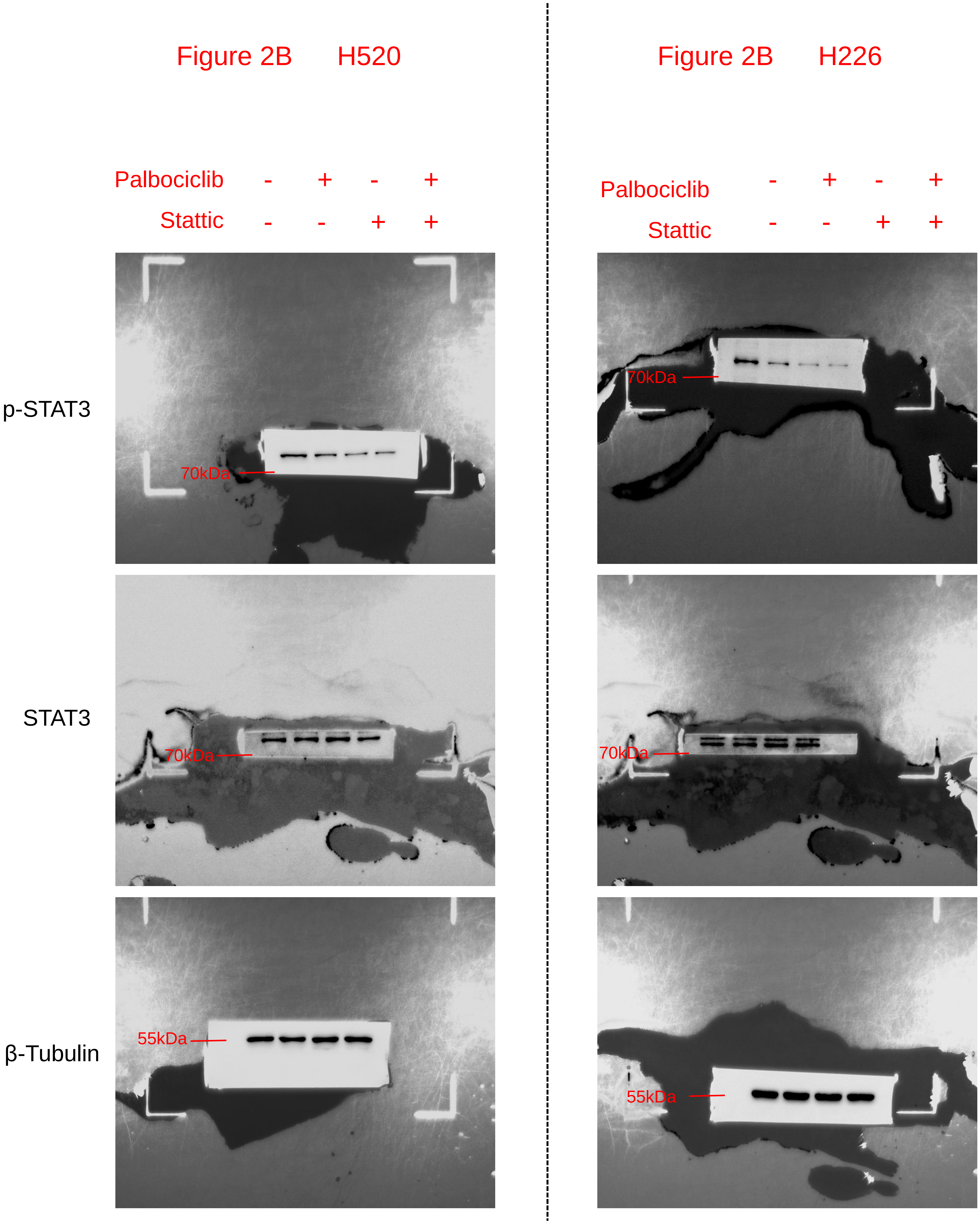

Figure S2. The original image Western blotting of Figure 2B.

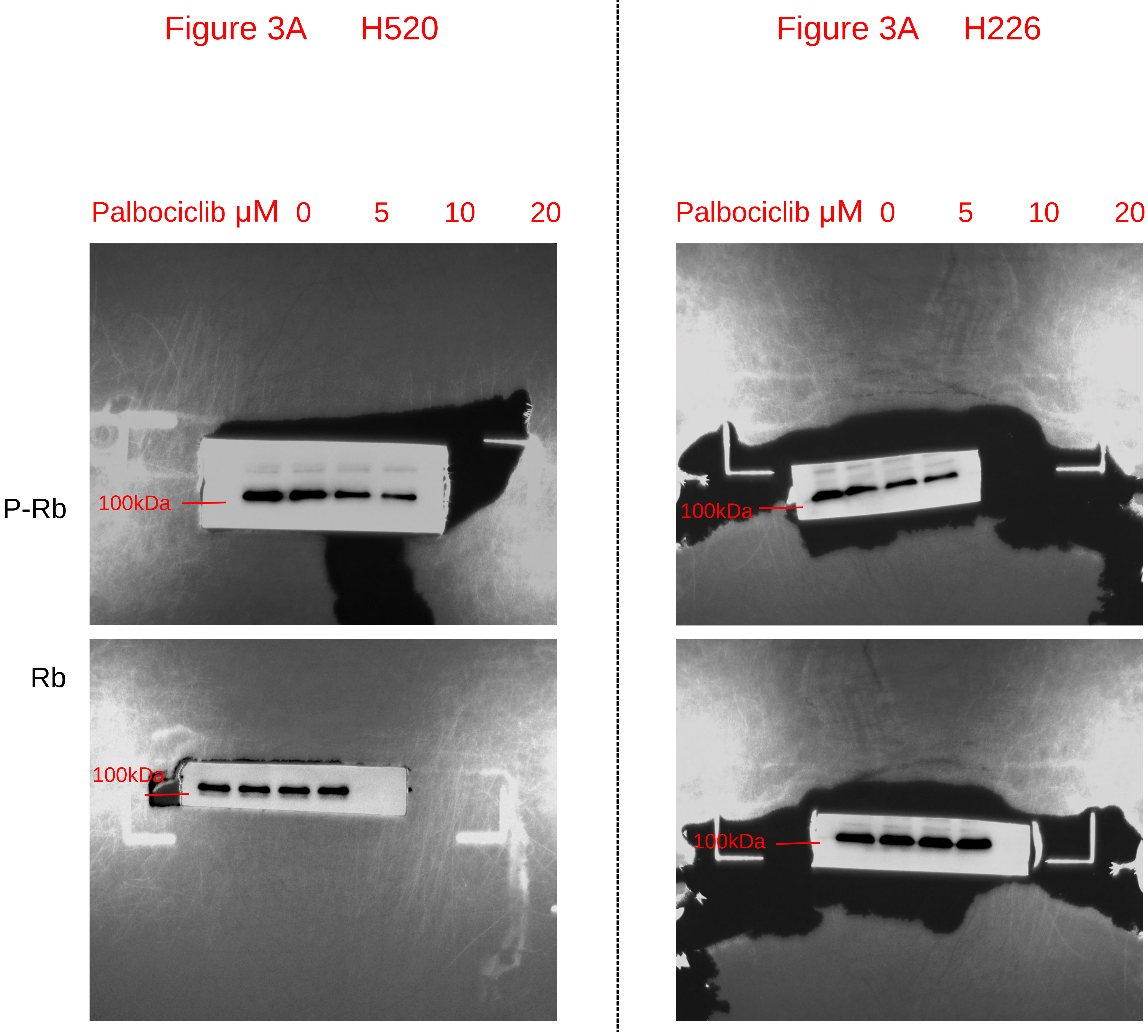

Figure S3. The original image Western blotting of Figure 3A.

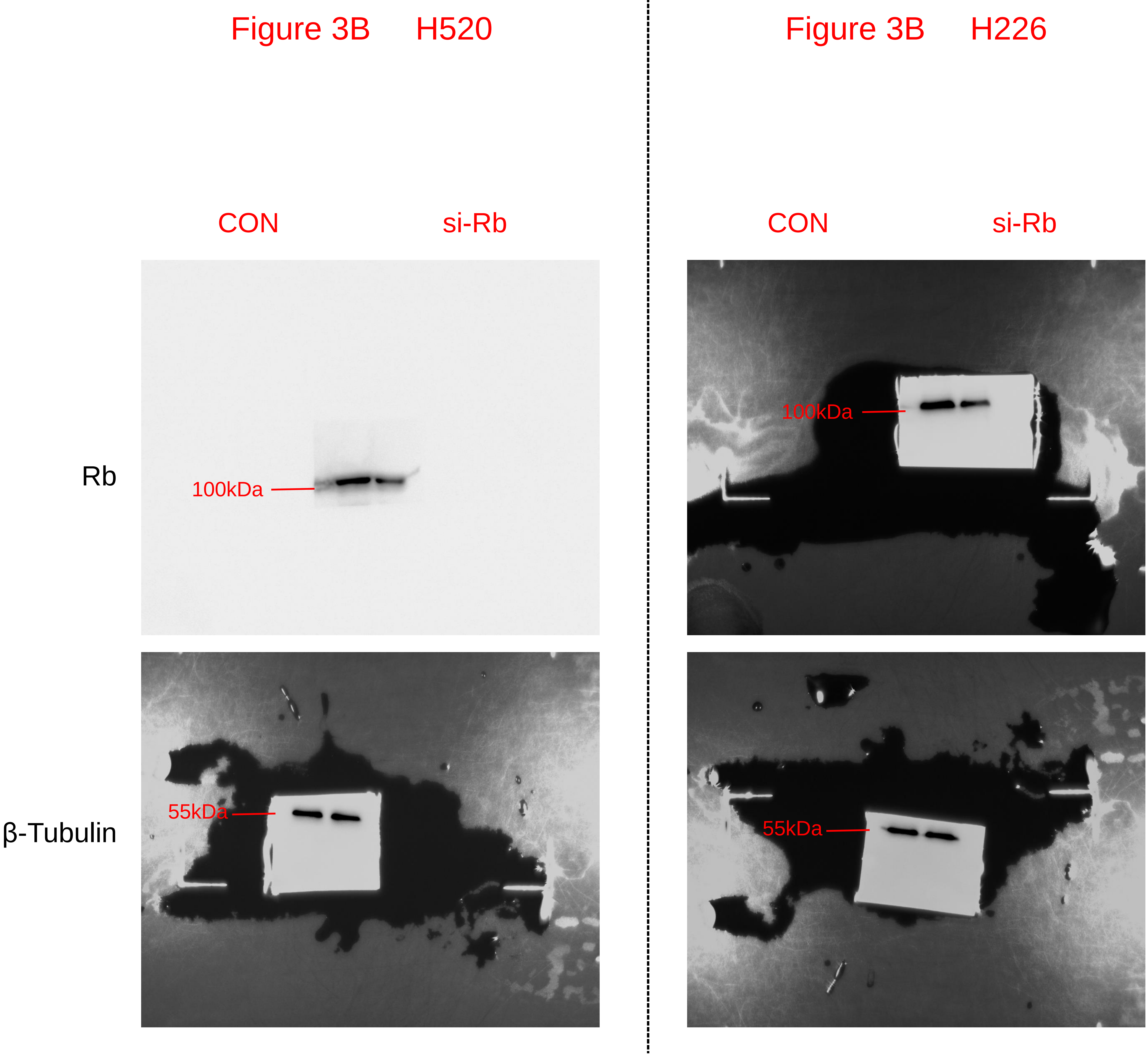

Figure S4. The original image Western blotting of Figure 3B.

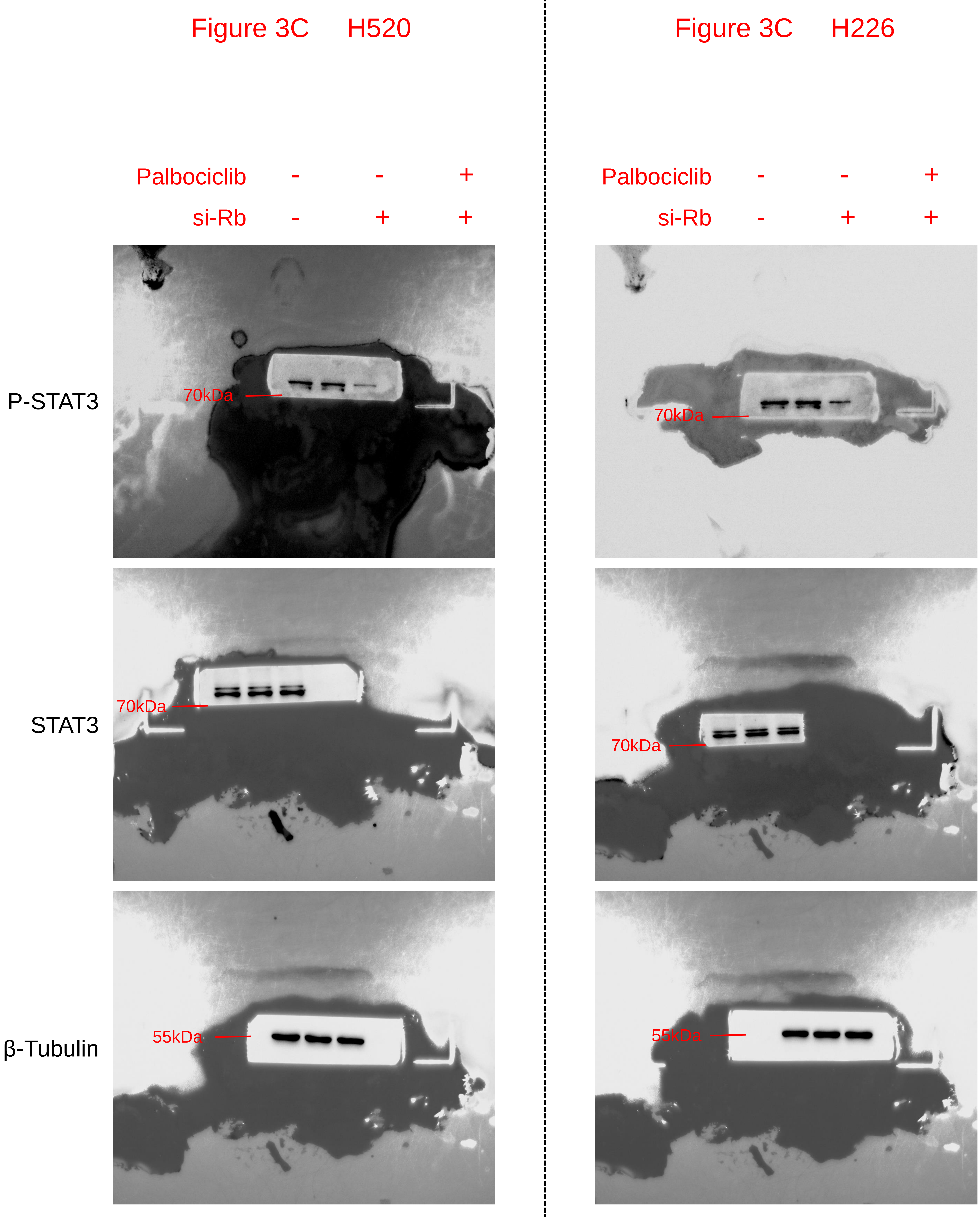

Figure S5. The original image Western blotting of Figure 3C.

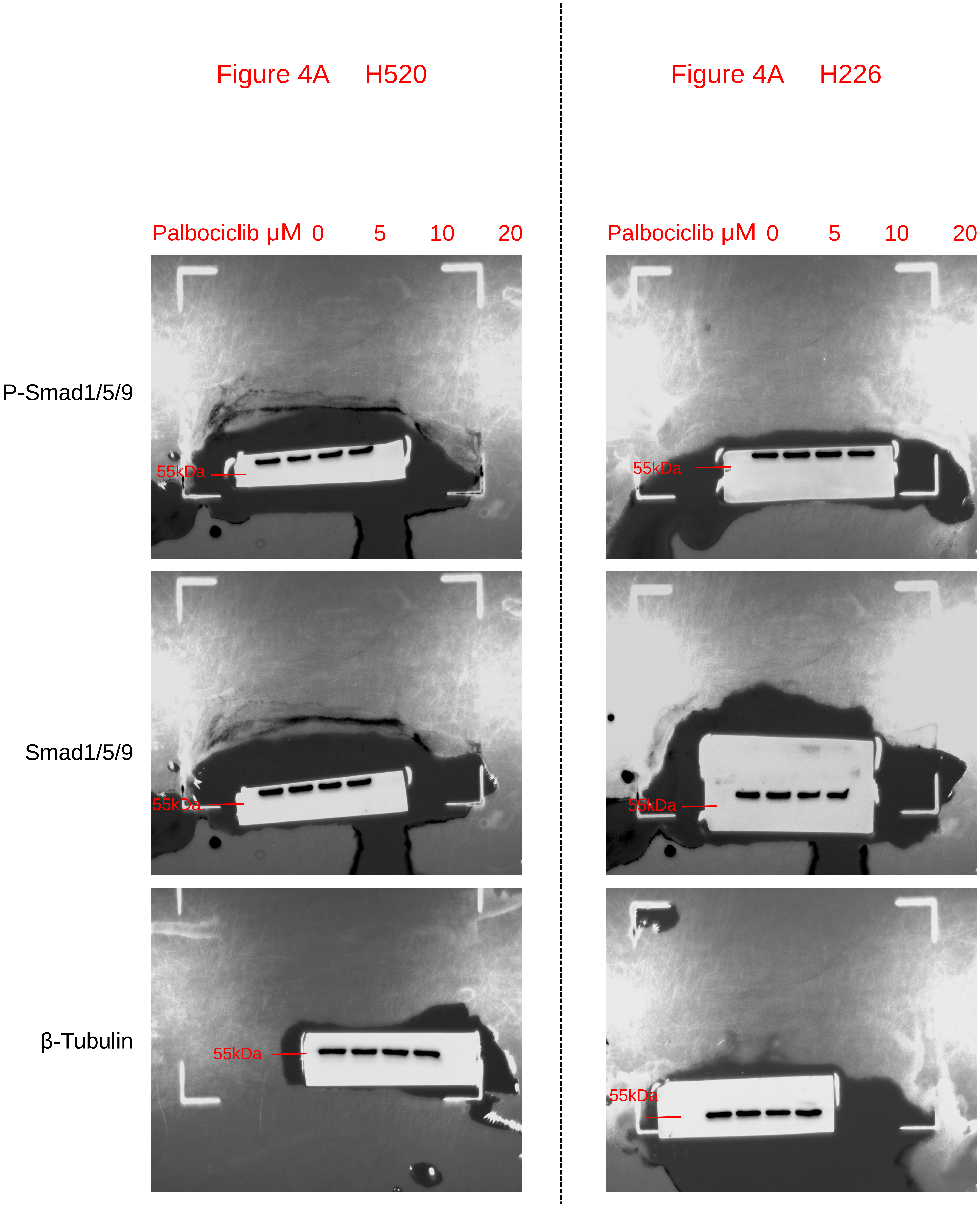

Figure S6. The original image Western blotting of Figure 4A.

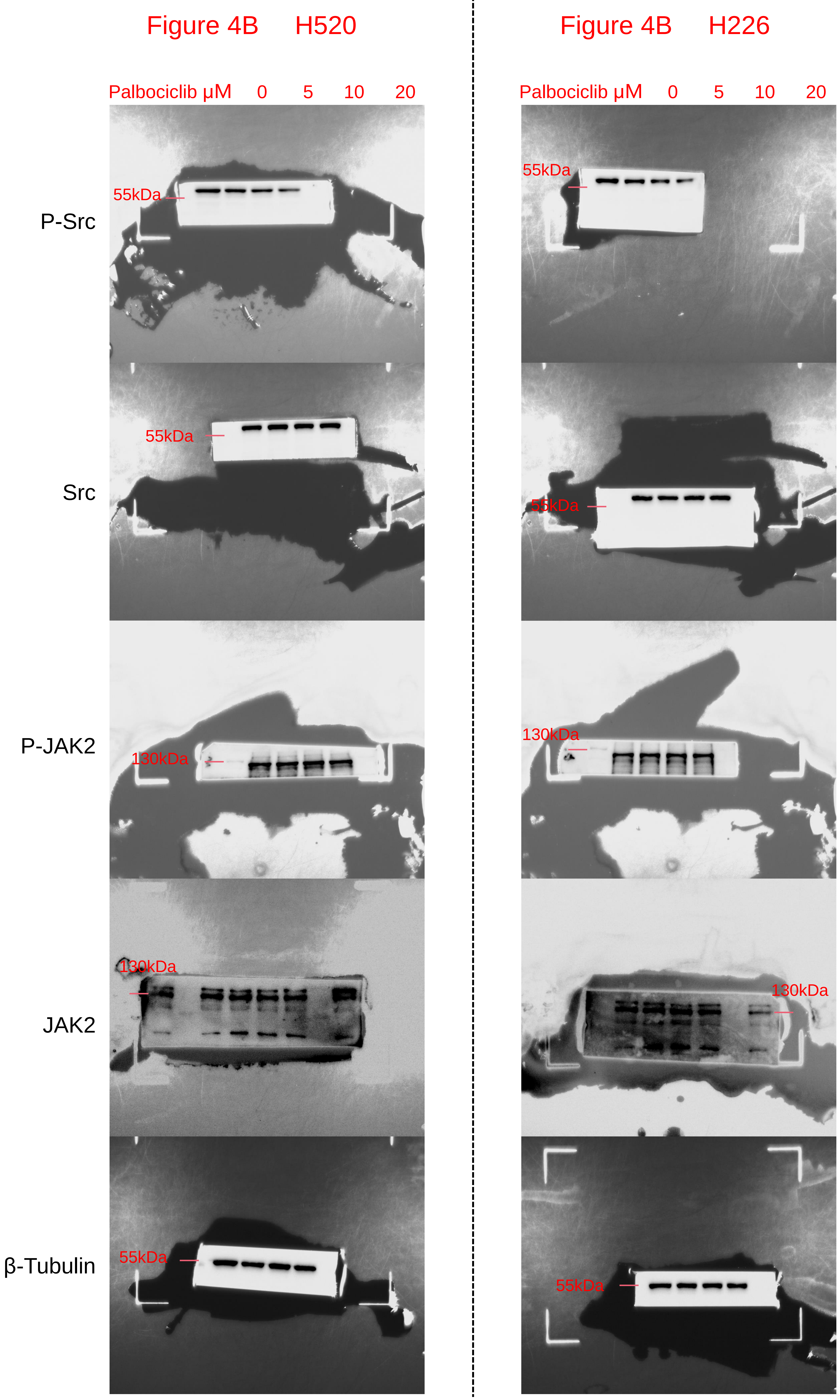

Figure S7. The original image Western blotting of Figure 4B.

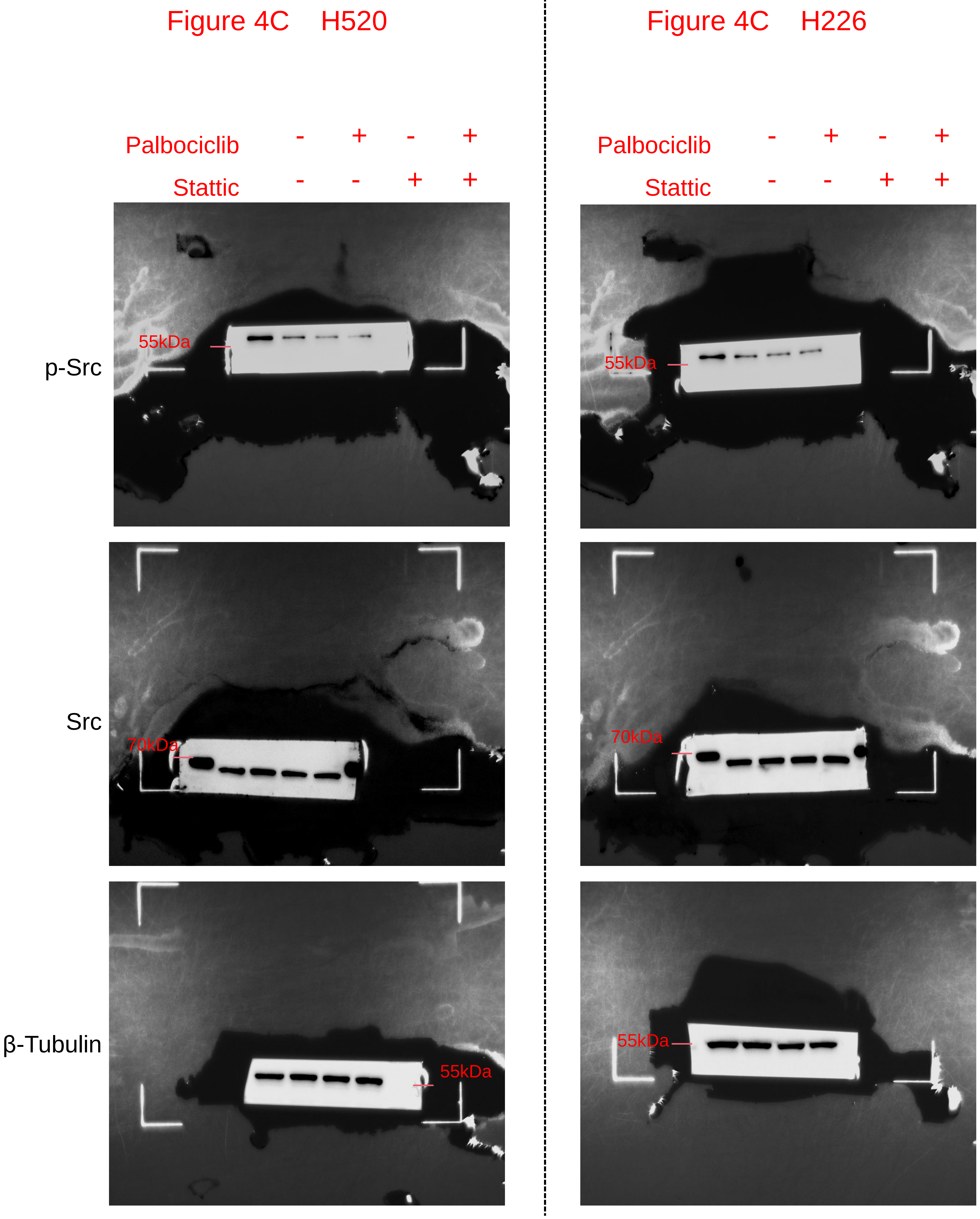

Figure S8. The original image Western blotting of Figure 4C.

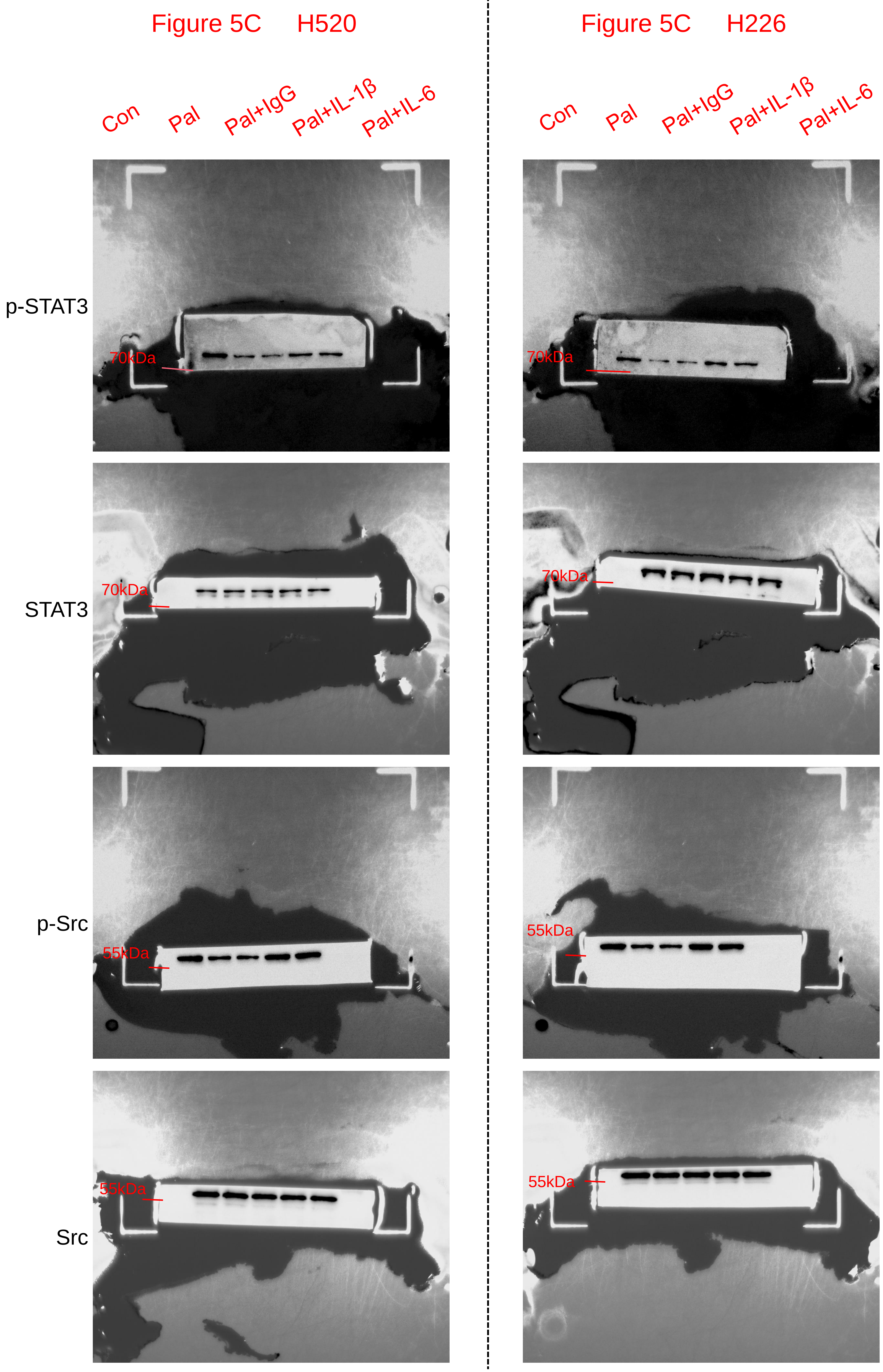

Figure 5C H520
Figure 5C H226
Con
Pal
Pal+IgG
Pal+IL-1β
Pal+IL-6
p-STAT3
STAT3
p-Src
Src
70kDa
55kDa

Figure S9. The original image Western blotting of Figure 5C.

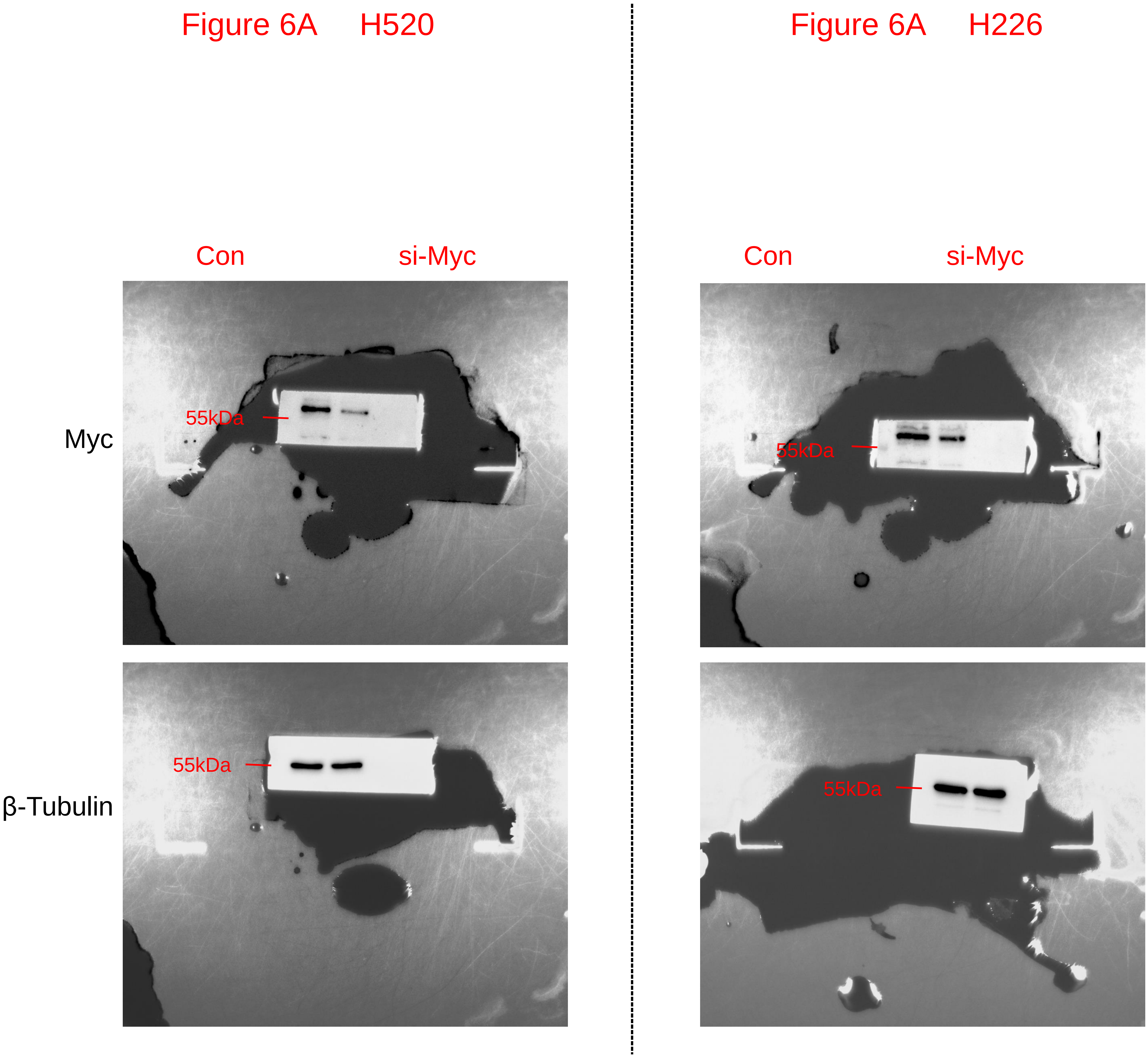

Figure S10. The original image Western blotting of Figure 6A.

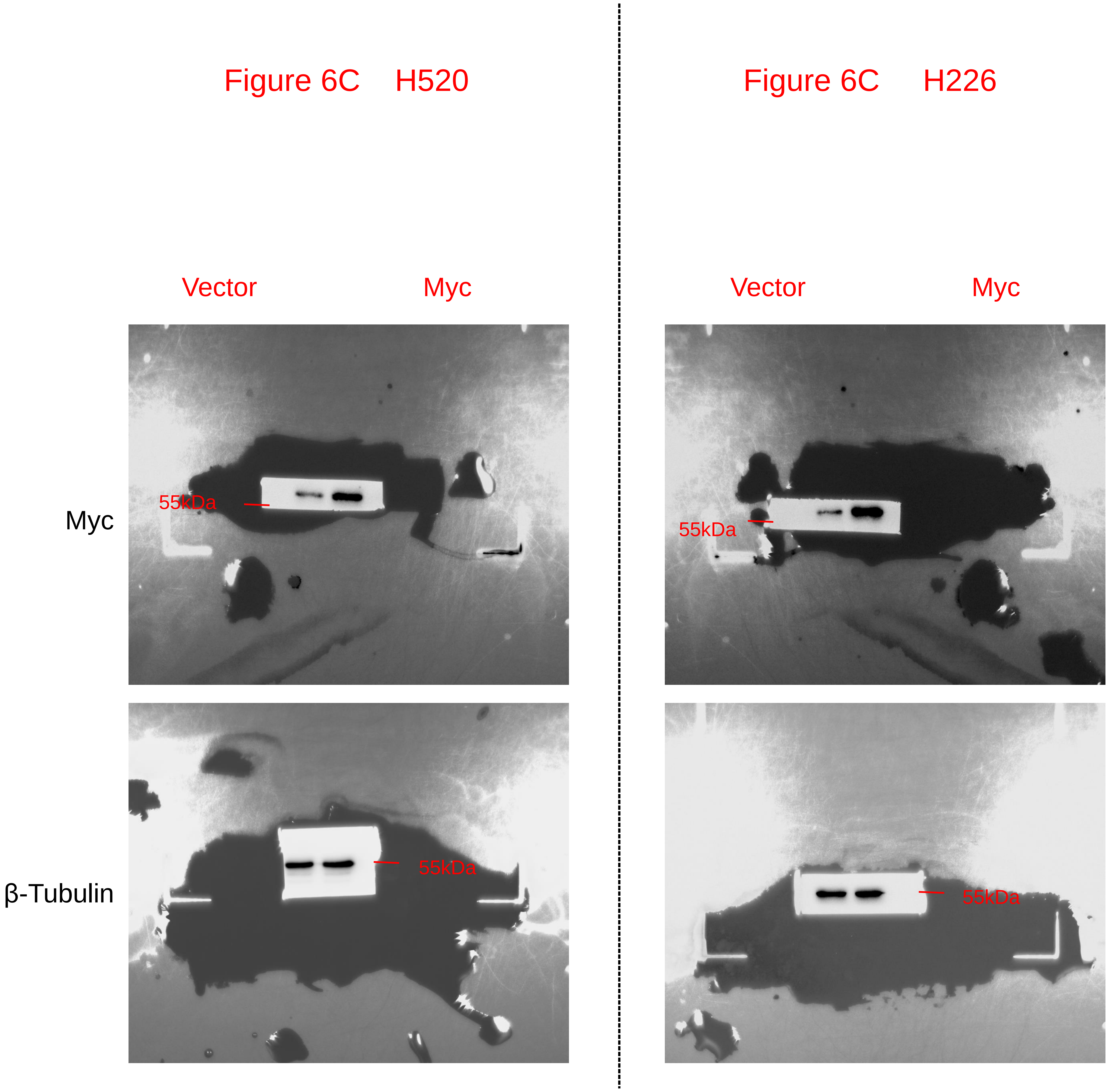

Figure S11. The original image Western blotting of Figure 6C.
